# Bullying as a Stressor in Mid-Adolescent Girls and Boys–Associations with Perceived Stress, Recurrent Pain, and Salivary Cortisol

**DOI:** 10.3390/ijerph15020364

**Published:** 2018-02-20

**Authors:** Viveca Östberg, Sara B. Låftman, Bitte Modin, Petra Lindfors

**Affiliations:** 1 Department of Public Health Sciences, Stockholm University, SE-106 91 Stockholm, Sweden; sara.brolin.laftman@su.se (S.B.L.); bitte.modin@su.se (B.M.); 2 Department of Psychology, Stockholm University, SE-106 91 Stockholm, Sweden; pls@psychology.su.se

**Keywords:** adolescents, bullying, cortisol, stress, victimization

## Abstract

Bullying involves repeated exposure to negative actions while also invoking a power asymmetry between the involved parties. From a stress perspective, being bullied can be seen as a severe and chronic stressor, and an everyday social-evaluative threat, coupled with a shortage of effective social resources for dealing with this particular stressor. The aim of this study was to investigate whether exposure to bullying among mid-adolescent girls and boys is associated with subjective and objective stress-related outcomes in terms of perceived stress, recurrent pain, and salivary cortisol. The data came from the School Stress and Support Study (TriSSS) including students in grades 8–9 in two schools in Stockholm, Sweden, in 2010 (study sample n = 392; cortisol subsample n = 198). Bullying was self-reported and measured by multiple items. The statistical analyses included binary logistic and linear (OLS) regression. Being bullied was associated with greater perceived stress and an increased risk of recurrent pain, among both boys and girls. Also, bullied students had lower cortisol output (AUC_G_) and lower cortisol awakening response (CAR_G_) as compared to those who were not bullied. Gender-stratified analyses demonstrated that these associations were statistically significant for boys but not for girls. In conclusion, this study demonstrated that being bullied was related to both subjective and objective stress markers among mid-adolescent girls and boys, pointing to the necessity of continuously working against bullying.

## 1. Introduction

Bullying has been recognized as a central stressor in the lives of school-aged children [1]. It is commonly defined as the repeated exposure to negative actions involving a power imbalance between a perpetrator(s) and a victim. The negative actions can be direct physical or verbal, such as hitting or teasing, and indirect, such as social exclusion [2,3]. It is not surprising that exposure to school bullying serves as a source of substantial stress, especially during the teenage period when peer relations become increasingly important. In the school context, membership in peer groups is vital for self-esteem and identity, while being the target of systematic harassments signals that the individual does not meet group standards [4]. Apart from being a threat to actual and perceived safety, bullying thus constitutes a social-evaluative threat.

With the repetition of negative actions over time being a core element of the definition of bullying, it also represents a chronic stressor. Indeed, earlier research has demonstrated that adolescents who reported having been bullied for a year or more rated the bullying as more stressful compared to those who reported exposure to harassments for a week or less [5]. While responses to short-term, or acute stress, can be considered adaptive, repeated, long-term, or chronic stress has been associated with detrimental health outcomes [6]. Adolescents who are bullied repeatedly, over a certain time period, have increased risks of stress-related health complaints [7,8,9] and of post-traumatic stress disorder (PTSD) [10,11] when compared to those who are not bullied.

Individual efforts to deal with stressors are commonly described in terms of coping, and the success in handling stressors is related to individual coping strategies and coping resources [12]. Different resources facilitate individuals’ coping with stressors, including the availability of social support [13]. However, the power imbalance characterizing bullying situations implies that individuals who are bullied have less access to social resources in the school setting. Indeed, students who are bullied are less likely to have friends at school than those who are not bullied [4,14]. Also, they are more likely to have poorer relations with their parents and teachers [4,15]. In addition, research has shown that individuals who are bullied tend to have no faith in supportive actions, which also means that they are less inclined to seek support [16]. Thus, being bullied can be viewed as a severe and chronic stressor and an everyday social-evaluative threat coupled with a shortage of effective social resources for dealing with this particular stressor.

Due to these characteristics, bullying exposure is likely to be related to activity within physiological stress systems. Both acute and chronic stress has been linked to the activity of the hypothalamic-pituitary-adrenocortical (HPA) axis and its output of the hormone cortisol. Typically, cortisol levels peak in the morning and decrease over the day, and earlier research has shown that morning cortisol and diurnal profiles of cortisol output are important measures of overall HPA-axis functioning [17]. While acute stress and the associated cortisol increases are adaptive, exposure to chronic stress is detrimental and may influence HPA-axis functioning [18]. For instance, distress caused by of social-evaluative threats has been shown to elicit cortisol responses [19], and the chronic stress of frequent and persistent peer rejection has been suggested to cause recurrent and chronic increases in cortisol among adults, thereby increasing the susceptibility for depression [20].

Few studies have investigated how exposure to bullying among adolescents relates to stress in terms of cortisol output. In a study of 12-year-olds, Vaillancourt et al. [21] showed that exposure to bullying was associated with higher mean levels of diurnal cortisol among boys but with lower levels among girls. Knack et al. [22] demonstrated that adolescents (fifth-to-eighth graders, mean age 12.2 years) who were bullied had lower levels of cortisol and a flattened cortisol awakening response when compared to those who were not bullied. Using sociometric data on exclusion and victimization by classmates among fourth graders (mean age 9.3 years), Peters et al. [18] found that socially excluded children had elevated cortisol levels at school, but lower morning cortisol and a flatter diurnal curve, when compared to those not excluded. The diurnal curves were less flat for excluded children with friends or high-quality friendships, suggesting a buffering effect of peer support [18]. Some studies have also compared cortisol output among bullied and non-bullied adolescents following an acute psychosocial experimental stress situation. A consistent finding of these studies was that bullied adolescents had a blunted cortisol response to the stress test compared to those who were not bullied [22,23,24,25]. This may indicate a HPA-axis dysregulation. Despite the mixed results, a number of studies suggest a lower rather than a higher cortisol output among bullied children due to a flatter diurnal curve. Vaillancourt et al. [21] suggest that a dampening of the HPA-axis may be adaptive in the case of bullying.

To gain a more thorough and overall view of stress among adolescents, it is recommended that researchers use multiple assessment methods and combine self-report measures and biomarkers of stress [1]. While salivary cortisol is a commonly used biomarker of stress, self-report indicators of stress include survey responses on perceived stress [26] as well as on recurrent pain, of which stress is a likely cause [27,28]. Accordingly, the aim of this study was to investigate whether exposure to bullying among mid-adolescent girls and boys is associated with both subjectively and objectively assessed manifestations of stress, namely perceived stress, recurrent pain, and the biomarker salivary cortisol.

## 2. Materials and Methods

### 2.1. Data

Data came from the School Stress and Support Study (TriSSS) conducted in the spring 2010. The project aimed to study school as an institution and links to stress and health (for additional details see Östberg et al. [29]). The Regional Ethical Board of Stockholm approved the project (ref. no. 2009/857-31/4). The study population included all students in grades 8 and 9 (14–16 years) in two elementary schools in Stockholm, Sweden (n = 545). Students with parental consent (455 students; 83%) were invited to participate on a voluntary basis. The project involved three parts: a questionnaire covering social relations, the school environment, stress, and health; saliva sampling; and individual semi-structured interviews. The present study used data from questionnaires and from saliva samples.

Students (n = 413; 76% response rate) completed questionnaires in the classroom. They subsequently sampled saliva at four time points during an ordinary school day: (1) immediately at awakening; (2) 30 min post-awakening; (3) 60 min post-awakening; and (4) at 20:00 h. During the sampling day, they also completed a diary with questions on time and date for saliva sampling, medication and chronic diseases, menarche (yes/no), and health behaviors.

Saliva was collected using plastic tubes (Salivette^®^) with a suspended insert containing a sterile cotton swab. Participants were instructed to chew on the swab, or keep it under the tongue for two minutes for it to become soaked with saliva, or actively spit directly in the tube. They were also instructed not to eat, smoke, consume coffee or tea, or brush their teeth 30 min before sampling saliva. Samples were stored at room temperature before being returned to the research team on the next school day, and then stored in a freezer (−20 °C) until analyzed.

Cortisol was determined using competitive radioimmunoassay (Spectria Cortisol RIA, Orion Diagnostica, Espoo, Finland; intra-assay precision <5%, 1.7–4.1% and inter-assay precision <10%, 4.3–9.0%). Each sample was analyzed twice and in randomized order with cortisol concentration expressed in nmol/L. Additional details regarding procedures for saliva sampling are reported elsewhere [29,30,31].

### 2.2. Measures

Bullied students were identified through the question: “Sometimes troublesome things happen at school. How often do the following things happen to you at school?” followed by four statements about experiences of actions/harassments, namely being socially excluded, subjected to disapproval, physically hit or hurt, and unjustly accused. The corresponding statements were: “No one wants to be with you”; “Other students show they don’t like you somehow, for example by teasing you or whispering or joking about you”; “One or more students hit you or hurt you in some way”; and “Other students accuse you of things you haven’t done or things you can’t help”. The response categories were: “Almost every day”, “At least once a week”, “At least once a month”, “Once in a while”, and “Never”. The statements cover verbal, indirect and physical harassments. The first three types correspond to commonly captured aspects of bullying [32]. The fourth type, to be unjustly accused, reflects victim-blaming as a fundamental aspect of bullying. This is a common strategy for justifying bullying which, at the same time, contributes to sustaining it [33,34]. Following a previously used operationalization [4,8,9], students exposed on a weekly basis to at least one of these harassment types were classified as being bullied.

Global stress was constructed from the question: “How often during the last six months have you had the following problems?” and the item “Felt stressed”. Those who reported being stressed every day or several times a week were categorized as stressed.

The Pressure and Activation Stress (PAS) Scale developed by Lindblad et al. [26] for elementary school students was used to cover two dimensions of stress. Pressure includes seven items: “I do too many things at the same time”; “I do not have time enough”; “I feel pressure from school demands”; “I feel pressure from demands at home”; “I feel pressure from my inner demands”; “I feel helpless”; and “I never feel really free”. Activation includes four items: “I rush even if I don’t have to”; “I eat rapidly even if I don’t have to”; “I keep a high speed all the day”; and “I find it difficult to relax”. Response categories were “Never”, “Seldom”, “Sometimes”, “Often”, and “Always” with ratings scored from 1 to 5. Each dimension was calculated as the mean value with the range 1–5 where higher values indicate greater stress. Internal consistency was high (Cronbach’s alphas: pressure = 0.84; activation = 0.77).

Recurrent pain was constructed from the question: “How often during the last six months have you had the following problems?” followed by the items: “Headache”, “Stomach-ache”, “Backache”, and “Pain in neck and shoulders”. The five response categories were “Every day”, “Several times a week”, “Once a week”, “Sometime during the month”, and “More seldom or never”. Recurrent pain was defined as having reported every day or several times a week for at least two of the items. The co-occurrence of pain indicates that stress is a likely cause [27].

Aggregate cortisol measures were calculated using the saliva samples collected at four time points during one school day. These were ‘Area under the curve with respect to ground’ (AUC_G_), measuring the total cortisol output during one day, using the three morning samples and the evening sample (i.e., samples 1–4); and ‘Cortisol awakening response with respect to ground’ (CAR_G_), measuring cortisol output during the first hour after awakening, using the three morning samples (i.e., samples 1–3). Raw, untransformed values of cortisol concentration and the trapezoid formula developed by Pruessner et al. [35] was used to calculate both AUC_G_ and CAR_G_. The formula for AUC_G_ is

$$AUC_{G}=\overset{n-1}{\underset{i=1}{\sum }}\;\frac{\left(m_{\left(i+1\right)}+m_{i}\right)\cdot t_{i}}{2}$$

where *t_i_* indicates the time distance between cortisol measurements, *m_i_* the individual measurement, and *n* the number of measurements [35]. With aggregate measures not being normally distributed these were log-transformed prior to the regression analyses.

### 2.3. Statistical Methods

Analyses of outcomes using questionnaire information were based on the study sample, i.e., all cases with valid information on the study variables (n = 392; 95% out of 413 students). Cortisol analyses included the subsample of students providing reliable salivary samples, information on sampling time, and taking the first sample within 15 min after waking (using a shorter time limit, i.e., 5 min instead of 15, do not change the study results) (n = 198). Linear (OLS) regressions, presenting unstandardized b coefficients with 95% confidence intervals (CIs) were performed for all continuous outcomes, while binary logistic regressions (displaying odds ratios with 95% CIs) were performed for dichotomous outcomes. In all analyses, gender and grade were adjusted for. In the analyses of cortisol, waking time, the time difference between waking and first sample, and menarche (for girls), were included as control variables.

## 3. Results

Table 1 presents descriptive statistics of both the study sample and the cortisol subsample. With the operationalization used, 13.5 percent of students reported being bullied at least weekly, with higher percentages among boys (21.9 percent) than among girls (7.8 percent). In the cortisol subsample, the percentages reported being bullied were lower, particularly among boys.

Table 2 presents results from analyses with exposure to bullying as the independent variable and global stress, pressure, activation, and recurrent pain as outcomes. Being exposed to bullying was clearly associated with an increased risk of perceived stress in terms of global stress (OR = 3.06, 95% CI 1.55, 6.03), pressure (b = 0.40, 95% CI 0.19, 0.62) and activation (b = 0.37, 95% CI 0.13, 0.62), as well as recurrent pain (OR = 3.39, 95% CI 1.62, 7.09). For all four outcomes, interactions between bullying and gender were tested, but none were statistically significant (data not shown). Gender-stratified analyses confirmed that the patterns were similar for girls and boys.

Table 3 includes findings from analyses of exposure to bullying and aggregate cortisol measures. In the analyses of the full cortisol subsample, being bullied was negatively associated with AUC_G_ (b = −0.30, 95% CI −0.53, −0.06), thus showing a lower total cortisol output during the day among students who were bullied compared with those who were not. Also, being bullied was negatively associated with CAR_G_ (b = −0.20, 95% CI −0.39, −0.02), with a lower awakening response among those who were bullied compared to those who were not. As a sensitivity test, nicotine use (yes/no) was included as a control variable, but did not substantially change the patterns of findings (data not shown). For both cortisol measures, we also ran models including the interaction term between bullying and gender. For CAR_G_ (but not for *AUC_G_*), the interaction was statistically significant (data not shown). The gender-stratified analyses showed that being bullied was significantly associated with AUC_G_ and CAR_G_ among boys, but not among girls. However, there was a tendency for bullied girls to have a lower total cortisol output (AUC_G_) than the non-bullied girls, although the association was not statistically significant.

**Figure 1:**
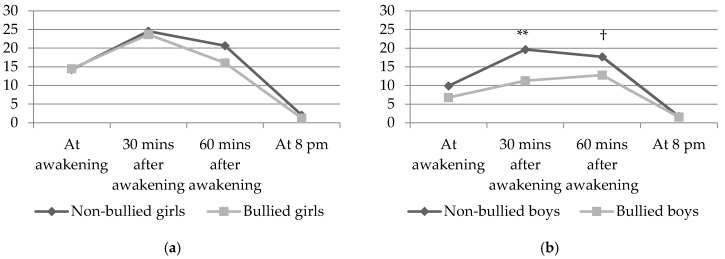
(**a**) Diurnal salivary cortisol output (nmol/L) at four time points during one day among non-bullied and bullied girls. n = 131; (**b**) Diurnal salivary cortisol output (nmol/L) at four time points during one day among non-bullied and bullied boys. n = 60. ** *p* < 0.01 ^†^ *p* < 0.10.

Figure 1a (girls) and Figure 1b (boys) show the mean values of salivary cortisol (in nmol/L) at the four time points during the day by exposure to bullying. Bullied students exhibited a flatter curve than the non-bullied students, thus indicating a lower cortisol output among the bullied. The difference between curves was larger among boys than among girls, reflecting the results from the regression analyses (Table 3). The mean values shown in the figures are presented in Table A1 in the Appendix A along with 95% confidence intervals, as well as b coefficients and *p*-values from gender-specific linear regression analyses of cortisol output for each of the four time points during one day.

## 4. Discussion

The present study investigated associations between exposure to bullying and a range of stress-related measures among mid-adolescent girls and boys, with the main contribution being the inclusion of both self-reports of stress and physiological outcomes in terms of cortisol output. The results showed that students who were exposed to bullying were more likely to report perceived stress and recurrent pain as compared to their non-bullied peers. Moreover, students who were bullied had lower total cortisol output (AUC_G_) and a lower cortisol awakening response (CAR_G_) than those who were not bullied. Gender-stratified analyses demonstrated that these associations were statistically significant only among boys. For total cortisol output, there was however a similar tendency among girls, although weaker and not statistically significant. This finding may relate to the sampling schedule of the present study asking both girls and boys to sample saliva at 30 and 60 min after waking. However, research suggests that post-pubertal girls are similar to adult women in exhibiting a delayed cortisol peak at 45 min after waking [36,37]. With the sampling protocol failing to capture a potentially delayed peak, this may obscure linkages among girls.

The substantial and statistically significant associations of exposure to bullying with perceived stress and recurrent pain were expected. They align with findings from previous studies which show that exposure to bulling is associated with an increased risk of health complaints [7,8,9] as well as an increased risk of post-traumatic stress disorder [10,11]. The results regarding cortisol output were also in line with some previous studies, for example the study by Knack et al. [22] showing that bullied adolescents had lower cortisol levels and a flattened cortisol awakening response than those who were not bullied. However, the findings of the present study extend previous research in also providing details regarding potential gender differences. Moreover, our findings reflect those of experimental studies including stress tests and demonstrating that bullied children had blunted cortisol responses compared with non-bullied children, indicating a dysfunction in the HPA-axis [22,23,24,25]. Thus, the present study findings align with previous research showing that exposure to bullying, as a chronic stressor, may distort the functions and the reactivity of the bodily stress system. The finding that bullying may have physiological effects is important, particularly in view of the research showing that blunted stress reactivity is associated with a range of adverse behavioral and health-related outcomes [38].

While the current study has several strengths, most notably the inclusion of both self-report and physiological stress measures, with cortisol being collected in a naturalistic, daily life, setting (with repeated saliva samples from one day), there are also limitations. The study included students in grades 8 and 9 (ages 14–16 years) in two elementary schools in Stockholm. Students in both these schools performed above the national average, and for both schools the proportion of students with parents having tertiary education was higher than the national average [29], thus limiting generalization to other groups. To corroborate our findings, studies including other age-groups and other sociodemographic characteristics are needed. However, it should be noted that the reported associations between exposure to bullying and recurrent pain have been shown in nationally representative samples as well [8,9]. Furthermore, collecting salivary cortisol in a naturalistic setting has merits but also puts demands on the participants. After inspection of the information noted in the sampling diaries, it was concluded that participants were well capable of adhering to the sampling protocol [29]. However, about half of the students had difficulties in keeping the 30 min time limit between breakfast and saliva sampling. This applied equally often to bullied and non-bullied students, thus suggesting lack of systematic bias. In addition, cortisol was only available for a subsample of students, and the percentages reporting to be bullied were smaller in this subsample than in the full study sample. In particular, boys who were bullied seemed less inclined to participate in the cortisol sub-study than the non-bullied boys. It is possible that this bias may have underestimated the associations between bullying and cortisol output. In any case, given the fact that the cortisol subsample only included eight boys who reported to be bullied, the results are somewhat tentative and should be interpreted with caution. To draw firm conclusions about the associations between being bullied and cortisol output, and particularly with respect to gender differences in these associations, studies using larger samples are needed.

The data used in the current study were cross-sectional, thus presenting a snapshot of students’ exposure to bullying and stress. Previous longitudinal studies have reported long-term consequences of bullying on various adverse health outcomes [3,4] but few prospective studies have investigated bullying and physiological stress markers later in life. Since our findings suggest a link between exposure to bullying and HPA-axis dysregulation, future research should address the potentially accumulated effects of bullying on bodily stress systems. For instance, earlier research has shown that a longer duration of bullying is experienced as more stressful than shorter durations of bullying [5], thus indicating that exposure to prolonged bullying has more detrimental effects on the stress systems. Accordingly, investigating the potential long-term bodily effects of bullying using prospective data seems like a promising avenue for future research.

## 5. Conclusions

Being bullied was related to several different stress measures among mid-adolescent girls and boys, including higher levels of perceived stress and recurrent pain, thus strengthening the overall conclusion that being bullied is associated with greater stress. For boys, we also found statistically significant associations between being bullied and lower cortisol output. For girls, a similar but slightly weaker and non-significant tendency was found for being bullied and total cortisol output. The finding that being bullied was related to a blunted cortisol output may be interpreted as a dysregulation within the HPA-axis, probably relating to chronic stress exposure. The clear associations between exposure to bullying and different subjective and objective stress measures among students make a strong argument for the necessity of continuously working against bullying.

## Figures and Tables

**Table 1:** Descriptives

**Study Sample**						
**Variables**	**All (n = 392)**		**Girls (n = 232)**		**Boys (n = 160)**	
	**n**	**%**	**n**	**%**	**n**	**%**
Bullied						
No	339	86.5	214	92.2	125	78.1
Yes	53	13.5	18	7.8	35	21.9
Global stress						
No	190	48.5	75	32.3	115	71.9
Yes	202	51.5	157	67.7	45	28.1
	**Mean**	**s.d.**	**Mean**	**s.d.**	**Mean**	**s.d.**
Pressure	3.06	0.81	3.31	0.68	2.71	0.85
Activation	2.87	0.89	3.10	0.79	2.53	0.92
	**n**	**%**	**n**	**%**	**n**	**%**
Recurrent pain						
No	325	82.9	180	77.6	145	90.6
Yes	67	17.1	52	22.4	15	9.4
**Cortisol Subsample**						
**Variables**	**All (n = 198)**		**Girls (n = 134)**		**Boys (n = 64)**	
	**n**	**%**	**n**	**%**	**n**	**%**
Bullied						
No	178	89.9	122	91.0	56	87.5
Yes	20	10.1	12	9.0	8	12.5
	**Mean**	**s.d.**	**Mean**	**s.d.**	**Mean**	**s.d.**
lnAUC_G_^a,b^	8.97	0.52	9.02	0.54	8.87	0.46
lnCAR_G_^b^	6.97	0.42	7.07	0.40	6.78	0.38

^a^ All n = 191; Girls n = 131; Boys n = 60. ^b^ nmol/L.

**Table 2:** Results from binary logistic regressions of global stress and recurrent pain, and from linear (OLS) regressions of pressure and activation, by exposure to bullying. Estimates are presented for bullied students (omitted reference category = not bullied).

Gender	Global Stress		Pressure		Activation		Recurrent Pain	
	OR	95% CI	b	95% CI	b	95% CI	OR	95% CI
All (n = 392)	3.06 **	1.55, 6.03	0.40 ***	0.19, 0.62	0.37 **	0.13, 0.62	3.39 **	1.62, 7.09
	Pseudo R^2^ = 0.13		R^2^ = 0.17		R^2^ = 0.12		Pseudo R^2^ = 0.06	
Girls (n = 232)	4.02 ^†^	0.90, 18.07	0.35 *	0.03, 0.68	0.36 ^†^	−0.02, 0.75	2.35 ^†^	0.86, 6.48
	Pseudo R^2^ = 0.02		R^2^ = 0.02		R^2^ = 0.02		Pseudo R^2^ = 0.01	
Boys (n = 160)	2.81 *	1.28, 6.17	0.43 **	0.11, 0.74	0.38 *	0.03, 0.72	5.15 **	1.70, 15.59
	Pseudo R^2^ = 0.04		R^2^ = 0.05		R^2^ = 0.03		Pseudo R^2^ = 0.09	

*** *p* < 0.001, ** *p* < 0.01, * *p* < 0.05 ^†^, *p* < 0.10. ^a^ Adjusted for gender and grade. ^b^ Adjusted for grade.

**Table 3:** Results from linear (OLS) regressions of cortisol output by exposure to bullying. Estimates are presented for bullied students (omitted reference category = not bullied).

Gender	lnAUC_G_		lnCAR_G_	
	b	95% CI	b	95% CI
All ^a^	−0.30 *	−0.53, −0.06	−0.20 *	−0.39, −0.02
	R^2^ = 0.10		R^2^ = 0.13	
Girls ^b^	−0.23	−0.56, 0.09	0.01	−0.24, 0.26
	R^2^ = 0.12		R^2^ = 0.01	
Boys ^c^	−0.33 ^†^	−0.68, 0.02	−0.51 **	−0.79, −0.23
	R^2^ = 0.15		R^2^ = 0.23	

** *p* < 0.01, * *p* < 0.05, ^†^ *p* < 0.10. ^a^ Adjusted for gender, grade, waking time, and time difference between waking and the first saliva sample. n = 191 (lnAUC_G_); n = 198 (lnCAR_G_). ^b^ Adjusted for grade, waking time, time difference between waking and the first saliva sample, and menarche. n = 131 (lnAUC_G_); n = 134 (lnCAR_G_). ^c^ Adjusted for grade, waking time, and time difference between waking and the first saliva sample. n = 60 (lnAUC_G_); n = 64 (lnCAR_G_).

## Appendix A

**Table A1:** Cortisol output (nmol/L) at four time points during one day. Mean values and 95% confidence intervals, and b coefficients and *p*-values from gender-separate linear regressions with non-bullied girls and non-bullied boys as the reference categories.

Gender	At Awakening				30 min after Awakening				60 min after Awakening				At 8 pm			
Girls	**Mean**	**95% CI**	**b ^a^**	***p***	**Mean**	**95% CI**	**b ^a^**	***p***	**Mean**	**95% CI**	**b ^a^**	***p***	**Mean**	**95% CI**	**b ^a^**	***p***
Non-bullied (n = 119)	14.29	12.35, 16.24			24.54	22.58, 26.51			20.66	18.66, 22.66			2.05	1.66, 2.45		
Bullied (n = 12)	14.50	8.95, 20.04	−1.91	0.550	23.68	18.00, 29.36	−0.83	0.805	16.04	9.13, 22.96	−4.08	0.234	1.23	0.76, 1.69	−0.882	0.183
Boys	**Mean**	**95% CI**	**b ^a^**	***p***	**Mean**	**95% CI**	**b ^a^**	***p***	**Mean**	**95% CI**	**b ^a^**	***p***	**Mean**	**95% CI**	**b ^a^**	***p***
Non-bullied (n = 52)	9.90	8.46, 11.35			19.65	17.62, 21.68			17.69	15.77, 19.61			1.72	1.18, 2.26		
Bullied (n = 8)	6.80	0.22, 13.38	−2.26	0.290	11.27	5.19, 17.36	−8.42	0.005	12.77	7.03, 18.52	−4.82	0.082	1.53	0.51, 2.54	−0.163	0.824

^a^ Regression analyses are adjusted for waking time and time difference between waking and the first saliva sample.
